# Using copper-foil explosions to generate underwater focusing shocks of different geometries

**DOI:** 10.1007/s00348-025-04155-1

**Published:** 2025-12-05

**Authors:** Sebastián Rojas Mata, Francesc Hernández Garcia, Michael Liverts

**Affiliations:** FLOW, Department of Engineering Mechanics, KTH Royal Institute of Technology, 100 44 Stockholm, Sweden

## Abstract

Focusing shocks are created underwater by exploding 10-$\mu$m-thick copper foils with circular and polygonal geometries. Their symmetry and trajectory are characterized to assess this technique’s potential contributions to fundamental and applied investigations of nonlinear wave propagation and high-energy-density phenomena. The foils are exploded using a pulsed power generator which delivers kiloamp currents in microseconds. Current and voltage time traces of the explosions are recorded concurrently with high-speed shadowgraph images of the shocks. The electric waveforms of the explosions of different foil geometries resemble each other, showing peak resistive voltages, currents, and powers around 10 kV, 300 kA, and 2.5 GW, respectively. By extracting the shocks’ trajectories through statistical analysis of the shadowgraph images, it is found that circular foils, whether free standing or attached to the inside of a plastic shell, create shocks which accelerate up to Mach 1.7. Comparable Mach numbers are achieved by exploding a circular wire array of 32 100-$\mu$m-diameter copper wires, indicating that foil designs perform similarly to this traditional design. In contrast, free-standing polygonal foils create shocks which travel at a constant near-sonic speed, seemingly behaving as non-interacting weak planar shocks. This contradicts the theoretically predicted reshaping and acceleration of such shocks; manufacturing imperfections are suspected to cause this unexpected behavior. Alternate designs in which foils are attached to polygonal plastic shells are tested and found to create shocks which do reshape and accelerate.

## Introduction

Extreme pressures and temperatures arise in materials when shocks converge on a small volume, a process called shock focusing (Apazidis and Eliasson 2019). This phenomenon forms the basis for devices serving diverse purposes, such as extracorporeal shock-wave lithotripsy (Lingeman et al. 2009; Cleveland and McAteer 2012; Neisius et al. 2015) or inertial-confinement fusion (Betti and Hurricane 2016; Perry et al. 1979; Shcherbakov 1983), as well as plays a role in natural systems spanning microscopic (bubble cavitation (Putterman and Weninger 2000; Plesset 1948)) to macroscopic (stellar-core collapse (Janka et al. 2007; Van Riper 1982; Burrows and Vartanyan 2021)) scales. Analytically describing shock focusing is a challenging task due to the nonlinearities arising in both the equations describing the shock propagation and the thermodynamic properties of media under such extreme conditions. Studies over several decades have investigated the trajectory, stability, and strength of focusing shocks in multiple dimensions and in different kinds of materials (see Chapter 3 in Apazidis and Eliasson (2019) for a review). Part of ongoing research aims to experimentally characterize how the shock’s geometry (e.g., circular v. polygonal in 2D or spherical v. polyhedral in 3D) and the medium through which it propagates affect its symmetry as it focuses. Such information is pertinent in the design of experiments conducting fundamental investigations as well as of devices with more applied objectives.

As captured by self-similar solutions for their trajectory (Guderley 1942; Hunter 1960; Van Dyke and Guttmann 1982; Brushlinskii and Kazhdan 1963; Lazarus 1981), focusing shocks accelerate due to shock–shock interactions which propagate concave converging geometries through the generation of Mach stems with increasing Mach number (Whitham 1957). While multiple simulations and experiments of focusing shocks in gas have verified and refined the theory (see Fig. 3.9 in Apazidis and Eliasson (2019) for a summary), similar work in water is more scarce. Circular shocks generated with underwater wire explosions seem stable and retain a high degree of symmetry up to focus (Krasik et al. 2016; Bland et al. 2017). Non-uniformities in these shocks are weakly unstable (Kozlov et al. 2013; Fedotov et al. 2007), which contrasts with how non-uniformities in analogous shocks in gas destabilize the shape and transform the shock into a polygon (Watanabe and Takayama 1991; Takayama et al. 1987). The focusing of polygonal underwater shocks remains unexamined, though both theory (Whitham 1957; Schwendeman and Whitham 1987) and experiments (Kjellander et al. 2010; Eliasson et al. 2007) show the peculiar evolution such shocks undergo in gas. The shock-shock interactions at the polygon’s vertices cause these to accelerate faster than the faces, thereby rounding and converting them into new faces. Thus, the original *n*-gon becomes a 2*n*-gon, which then continues reshaping into a new *n*-gon as the original faces become new vertices. This cycle repeats, rotating the polygon as it focuses. Whether the same behavior occurs with underwater shocks has not been verified experimentally, potentially due to the challenge of creating underwater polygonal shocks.

In this paper, we explore the feasibility of exploding thin copper foils to generate focusing underwater shocks of different geometries. Although thin-foil explosions have been used for collision welding (Vivek et al. 2013; Hasegawa et al. 2024), dielectric flyer acceleration (Chace and Moore 1962; Asmedianov et al. 2025), and fusion (Slutz et al. 2016; Shelkovenko et al. 2020a), their application to shock generation is more limited (Mihara et al. 2009; Asmedianov et al. 2023); the preceding works exploded circular arrays of wires to generate their shocks. The technique works by pulsing a high current through the foils or wires so they heat up, undergo multiple phase transitions, and violently expand, thereby serving as pistons to compress the surrounding water (Taylor 1950; Krasik et al. 2008; Shi et al. 2021). While wire-array explosions are a tried-and-true approach, thin-foil explosions may provide an equally effective alternative with more geometrical freedom. Therefore, we build and test several proof-of-concept thin-foil designs to gain initial insight into the quality and behavior of the resulting shocks. We first describe in Sect. 2.1 the high-power facility used to generate underwater shocks along with the accompanying diagnostics for discharge characterization and flow visualization. The novel thin-foil designs which we implement into this facility follow in Sect. 2.2. In Sect. 3, we present the results of the tests, providing both qualitative assessments of the generated shocks’ shapes and statistical characterizations of the shocks’ position and velocity. We discuss the performance of our designs as well as conduct preliminary tests of improved designs in Sect. 4, ending with concluding remarks in Sect. 5.

## Experiment

We first provide a brief overview of the experimental facility used to generate and characterize underwater shocks; full details of the system are available in Hernández Garcia et al. (2025). The test cells described in Sect. 2.2 build on experience acquired during initial studies of shock generation by single-wire explosions.

### Generating and characterizing underwater shocks

**Fig. 1:**
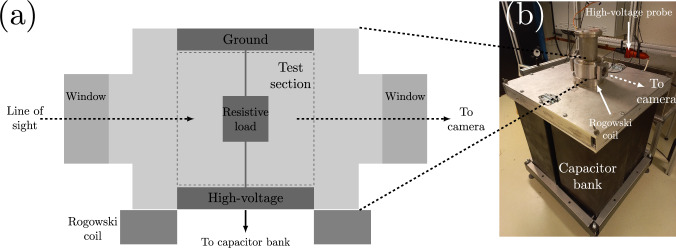
**a** Aluminum test chamber with 100 mm inner diameter. The high voltage side connects to the capacitor bank, while the ground side connects to the chamber’s body. **b** Test chamber sitting on top of the capacitor bank. The trigger circuit (not visible) sits behind the capacitor bank

To create shocks underwater, we use kiloamp currents arising during the rapid discharge of a pulsed-power generator (PPG) to explode resistive loads placed inside a test chamber filled with water. The PPG consists of a 10 $\mu$F capacitor bank which a 3 stage Marx generator triggers and discharges over tens of microseconds. Carefully tuned spark gaps in both apparatuses ensure safe and repeatable operation of the PPG. We measure the total voltage drop *V* across the resistive load with a high-voltage probe connected to the capacitor bank’s high-voltage electrode; a Rogowski coil encircling this electrode provides us with the current *I*. As shown in Fig. 1, the test chamber sitting atop the capacitor bank has two windows providing optical access to the test section. We can connect different resistive loads inside this test section to the high-voltage and ground sides of the capacitor bank. The PPG’s rapid discharge provides the high current needed to turn the resistive load into a piston to compress the surrounding water.

To visualize the shock created by this exploding piston, we employ a modified *z*-type shadowgraph configuration (Settles 2001) comprising a 500 W, 690 nm pulsed laser for backlighting and a high-speed camera for recording $400\times 250$-pixel frames at a rate of 5 Mfps (i.e. 200 ns resolution). The frame exposure is 110 ns, 20 ns of which the laser pulse illuminates, which means that shock fronts traveling between 1.5-3 km/s (Mach 1–2) would smear over 30–60 $\mu$m. However, our spatial resolution of $\sim$110 $\mu$m/pixel dominates the measurement uncertainty, so this smearing effect impacts our measurements negligibly. The modification in the *z*-type configuration consists of an additional corrector lens placed in front of the camera to improve focusing quality (Torres et al. 2023) given the spatial constraints of the lab. We synchronize the PPG’s discharge and the camera’s recording with a common trigger signal to ensure that we capture the relevant dynamics within the 51.2 $\mu$s (equivalent to 256 frames) available at this frame rate. Section 3.2 describes how we process these shadowgraph images to determine shock positions and velocities.

### Creating focusing shocks of different geometries

**Fig. 2:**
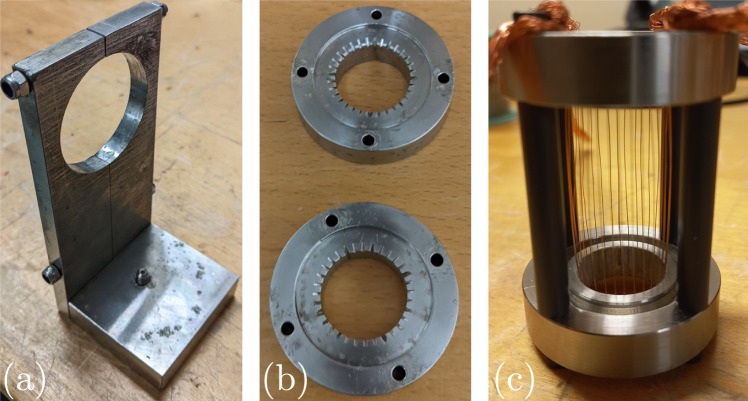
**a** Stainless steel test-cell stand which sits on the high-voltage side inside the test chamber. **b** Stainless steel electrodes with wire grooves. The inner and outer diameters are 20 mm and 40 mm, respectively, while the thickness is 10 mm. **c** Assembled test cell with a circular wire array

Many studies have placed a thin metal wire as the resistive load in facilities similar to ours to investigate a variety of physical phenomena, ranging from shock dynamics (Bennett 1958; Chace and Moore 1962; Krasik et al. 2008) to plasma instabilities (Oreshkin 2008; Oreshkin and Baksht 2020) to nanoparticle production (Sen et al. 2003; Sindhu et al. 2008). The explosion of a single wire creates a cylindrical shock which weakens as it expands; using this as a building block, arrays of many wires can be designed so that the individual shocks coalesce into a composite geometry, for example, a plane (Maler et al. 2022; Strucka et al. 2023), a converging circle (Efimov et al. 2008; Krasik et al. 2016; Bland et al. 2017), or even a converging sphere (Antonov et al. 2013). This established method serves as a benchmark against which to compare our foil designs, so we implement it into our system with the components pictured in Fig. 2. The test cell contains a 20-mm-diameter circular array of 32 wires, each 100 $\mu$m in diameter and 50 mm in length. Once clamped to the stand, the test cell sits inside the test chamber such that its axis aligns with the camera’s line of sight, allowing us to see the single-wire shocks coalesce into a focusing shock.

**Fig. 3:**
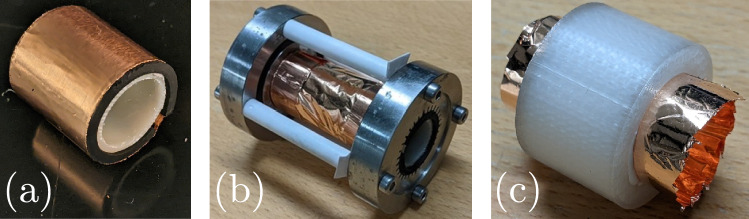
**a** Circular adapter for connecting foil to the test cell’s electrodes. **b** Assembled test cell with a free-standing circular foil 20 mm both in diameter and length. **c** Circular foil glued to the inside of a cylindrical shell. The shell is 20 mm long with inside and outside diameters of 20 mm and 30 mm

**Fig. 4:**
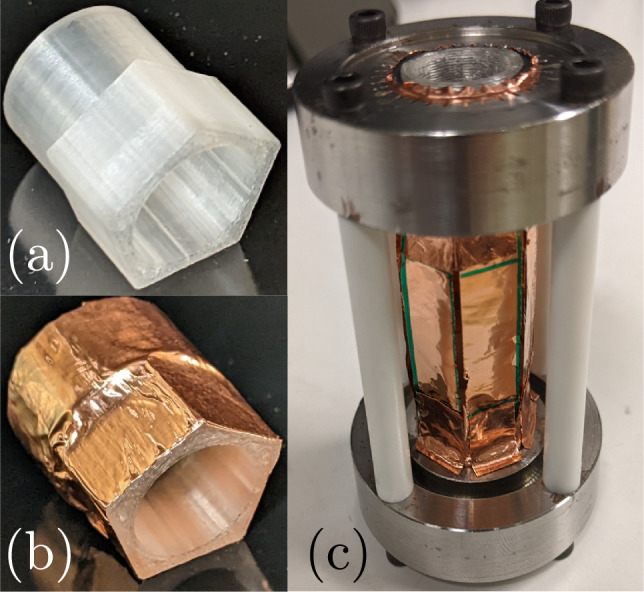
Circle-to-pentagon adapter **a** without and **b** with copper tape. **c** Assembled test cell with a hexagonal foil 20 mm in length. The press fit adapters and copper tape ensure uniform electrical connection

Wire-array designs require enough time and space for the individual wires’ shocks to merge into the desired geometry. Spatial constraints or relative delays in the wires’ explosions may prevent proper coalescing before focusing, whereas exploding a suitably shaped foil would generate the desired shock geometry from the start. This outcome relies on the foil exploding uniformly, which seems to be the case down to scales of tens of $\mu$m (Baksht et al. 2015; Shelkovenko et al. 2018, 2020b); however, there is a risk that the subsequent shock’s shape may degrade (Asmedianov et al. 2023; Hasegawa et al. 2024). As shown in Fig. 3, we press fit conductive adapters into the electrodes and mount a circular copper foil by using 35-$\mu$m-thick conductive copper tape to attach 10-$\mu$m-thick foil to these adapters. The electrodes are 40 mm apart and each adapter only extends 10 mm into the space between them, yielding a 20-mm-long, 20-mm-diameter free-standing foil cylinder in the middle of the test cell. Though carefully mounted, this free-standing foil can have small wrinkles which may lead to wrinkled shocks. We therefore also test a similar design in which the foil is glued to the inside of a cylindrical shell as a way to prevent such wrinkling. Finally, by using adapters with different shapes at each end, we can similarly mount strips of foil to create polygonal geometries as pictured in Fig. 4. The free-standing polygonal foils are also 20 mm long, and their circumscribing circle is 20 mm in diameter, making them as close in size to the circular foils as possible.

## Results

Using the designs described above, we characterized the explosions and resulting shocks of a benchmark wire array (circle), three free-standing foils (circle, pentagon, and hexagon), and a shell-attached foil (circle). We varied the discharge voltage of the PPG’s capacitor bank between 22.5 and 23 kV so that all configurations had the same initial available electrical energy-per-mass of 23.5 kJ/g. Along with the similar dimensions of the designs, this ensures that any differences in the resulting shocks derive from geometry or the quality of the foil or wire explosions.

### Foil explosions and shock quality

**Fig. 5:**
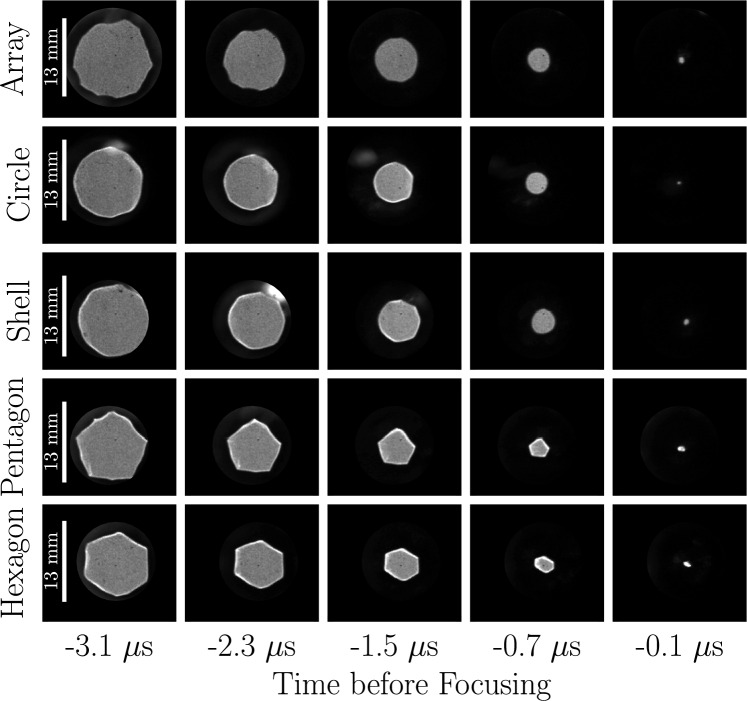
Time sequences of shadowgraph images for focusing shocks generated by underwater explosions of a circular wire array (first row), a free-standing circular foil (second row), a shell-attached circular foil (third row), a free-standing pentagonal foil (fourth row), and a free-standing hexagonal foil (fifth row)

We present in Fig. 5 time sequences of shadowgraph images of the focusing shocks generated by the different geometries. The strong density gradients behind the shocks deflect away all laser light, producing easily discernible shock fronts separating the undisturbed (gray) and post-shock (black) water. As the shocks focus some time between the last frame with an identifiable shock front and the next all-black frame, the time of focus has an uncertainty of up to 200 ns. Thus, we take the conservative approach of defining the time of focus as 100 ns after the last frame with a visible shock; this provides a common reference across all tests. First to note is the great symmetry of the shocks, which indicates that the foils exploded and expanded uniformly along their circumference. In separate tests not shown, we rotated the test cells by 90$^{\circ }$ to view them from the side and verified that the foils also explode uniformly along their length. We see that small perturbations (e.g., from the wire-array shocks meeting) eventually disappear, whereas larger perturbations (e.g., from the foils’ wrinkles in the pentagon’s top-right face) persist for longer times/distances. Far away from the focus center, smoothing corrugation effects overcome destabilizing convergence effects to eliminate short-wavelength perturbations, but for long-wavelength perturbations the pressure behind the corrugation is not enough to correct the shape before the convergence effects become comparable (Kozlov et al. 2013). Regardless of these minor imperfections, the images demonstrate that foil explosions successfully produce the desired geometries from the start. Both the free-standing and shell-attached circular foils create more circular shocks at the beginning than the wire array, yet the shapes of all three circularize as they focus. The pentagonal and hexagonal shocks maintain their shape up to focusing, a behavior which differs to the expected doubling of sides and rotation discussed in Sect. 1; we discuss this further in Sect. 4.

**Fig. 6:**
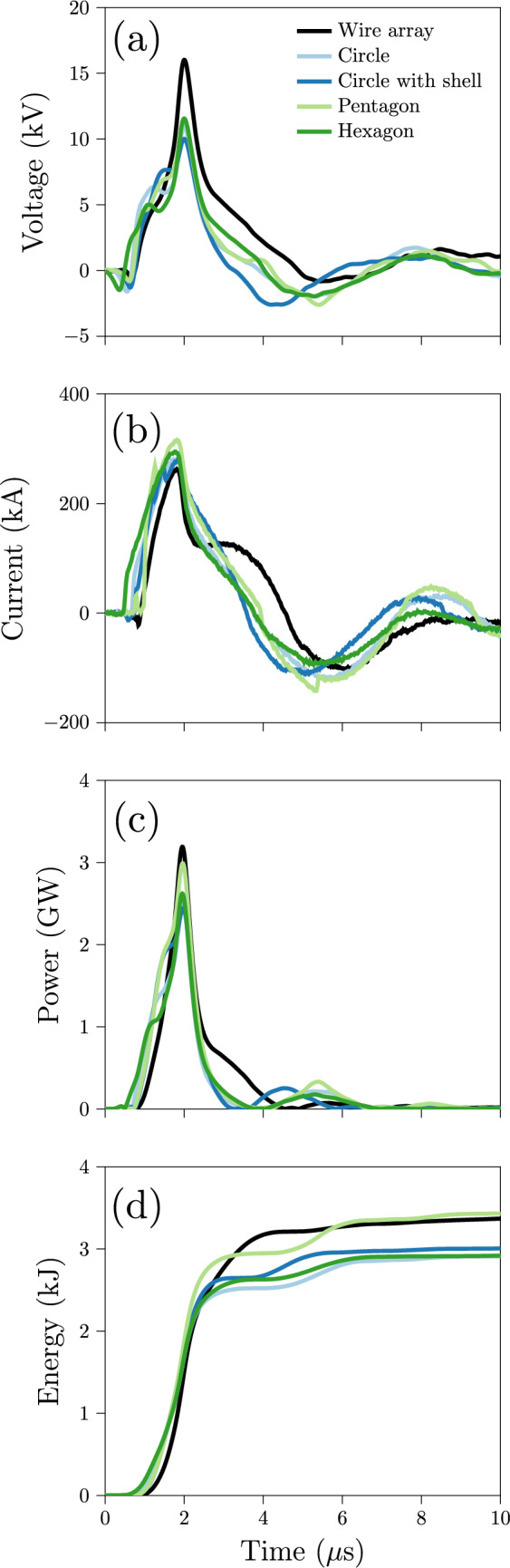
Time traces of the **a** resistive voltage, **b** current, **c** instantaneous power, and **d** cumulative electrical energy delivered for the discharges of the different geometries. We reference time so that the peak in resistive voltage occurs at 2 $\mu$s

Figure 6 shows the time traces of the discharges’ electrical parameters with times referenced such that the peaks in resistive voltage $V_{\text {R}}$ occur at 2 $\mu$s. This voltage, rather than the total voltage *V*, informs about the energy transfer to the resistive loads. It is given by $V_{\text {R}} = V - L\frac{{\text {d}}I}{{\text {d}}t}$, where *L* is the inductance of the wire array or foil cylinder. Although *L* varies nonlinearly in time, we assume it is constant for an initial comparison of the different discharges. We use analytical expressions (Rosa 1908) for these inductances as they are too small ($\sim$14 nH) to measure with standard RLC meters. Analytical expressions for the polygonal prisms are not available, so we approximate their inductance with that of the cylinder to enable an initial rough comparison between designs. We also calculate the instantaneous power $P = IV_{\text {R}}$ which we integrate in time to determine the cumulative electrical energy delivered to the resistive load during the discharge. The foil explosions closely resemble each other but do not feature the current restrike observed in single-wire explosions (Vlastós 1968; Chung et al. 2016) which the wire-array discharge somewhat exhibits around 2–4 $\mu$s. This discharge also reaches a higher peak voltage; however, its lower current leads to similar power and energy traces as for the foil discharges. Finally, the small secondary bumps in power and energy deposition of the foil discharges (around 4–6 $\mu$s) indicate that these discharges are less damped than that of the wire array.

Though beyond the scope of this work, a dedicated analysis comparing the explosions of open and closed foil configurations to those of wires would contribute to ongoing research (Baksht et al. 2015; Shelkovenko et al. 2020a, b) on the mechanisms involved in thin-foil explosions and their fundamental differences from wire explosions. These differing dynamics impact the efficiency of shock generation along with factors such as how cylindrical (wire) and planar (foil) geometries direct more or less energy in the direction of converging water flow. Additional analysis of the electrical discharges (for example, by considering the specific current action integral Oreshkin et al. 2007; Asmedianov et al. 2024) would provide guidance for improving the energy deposition into the explosion, as has been done for wire-array designs (Krasik et al. 2008, 2016; Bland et al. 2017). In the meantime, these foil designs successfully provide the experimental means to investigate the dynamics of underwater focusing shocks.

### Statistical characterization

**Fig. 7:**
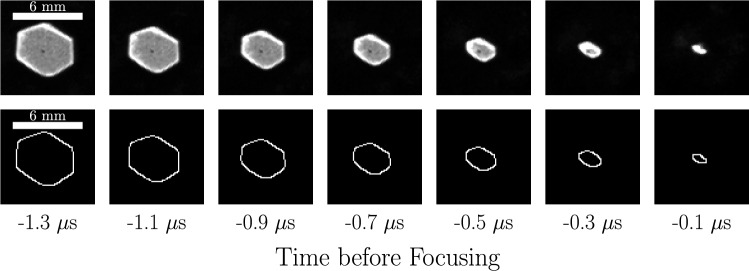
Time sequences of the hexagonal focusing shock’s shadowgraph images (top row) alongside the images’ detected edges (bottom row). While not perfectly symmetric, the hexagonal shape persists up to focus

We now describe our approach to quantitatively measure shock position and velocity. Instead of fitting shapes onto the shadowgraph images, we adopt a statistical approach involving distributions of shock position. These provide us with more flexibility for quantifying variabilities due to shape imperfections than fits. This requires us to identify the pixels corresponding to the shock front in the shadowgraph images. A simple Canny edge detector (OpenCV 2025) followed by minor manual refinements to remove residual noise produces images such as those shown in Fig. 7 for the case of the hexagonal foil.

**Fig. 8:**
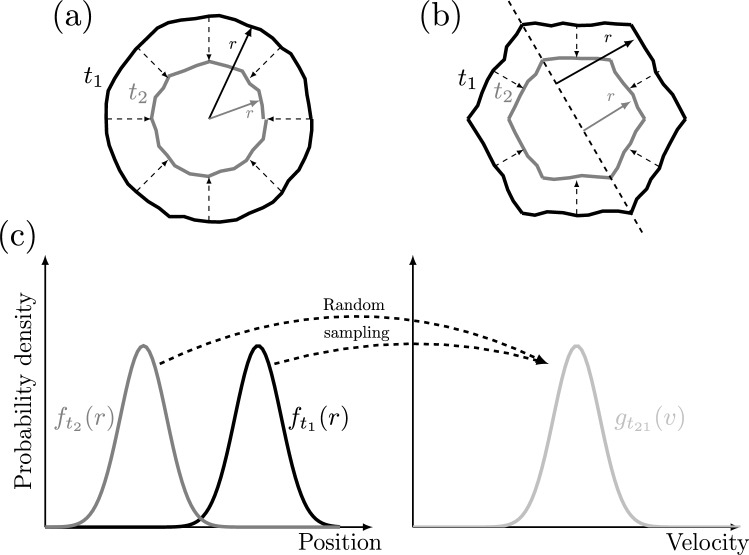
Definition of the position *r* for **a** circular and **b** polygonal focusing shocks at times $t_1$ and $t_2$. The focus center serves as the origin. **c** The shock fronts are not perfectly symmetric, so we construct position PDFs $f_{t_1}$ and $f_{t_2}$, respectively. We combine random samples of these PDFs to create the velocity PDF $g_{t_{21}}$

We define the focus center as the center-of-mass of the edge pixels in last frame with a visible shock. With this as our coordinate system’s origin, we then measure the local position *r* of the shock front’s pixels for all frames in the sequence. However, as illustrated in Fig. 8, different definitions of position apply to the different geometries. For a circular shock (panel a), position is just the radial distance from the origin. For a polygonal shock (panel b), we use the perpendicular distance from each face of the polygon to the corresponding parallel line passing through the origin. We combine these measurements to construct probability distribution functions (PDFs) of shock position $f_t(r)$ for each time *t* (panel c). By randomly sampling the position PDFs of two (or more) frames, we can also construct PDFs for the shock velocity $g_t(v)$ by pairing up the samples and calculating the time derivative using a second-order central difference formula (Moin 2010). Thus, the velocity PDF for a frame depends on the position PDFs of the frames before and after. This means that we do not calculate a velocity PDF for the first or last frame; a one-sided difference formula could be applied, but such approach inspires lower confidence than the central difference formula.

To report averages and variabilities of the PDFs, we use medians and median absolute deviations

1 $$\begin{aligned} \text {MAD} = \text {median}(|r_i - \text {median}(r_i)|). \end{aligned}$$

Note that imperfections in shape, rather than measurement uncertainty (i.e., the camera’s spatial resolution), constitute most of the position PDFs’ variability, except for very close to focus. These imperfections, which are more pronounced early on, lead to greater overlap in the position PDFs due to the discrete nature of the pixels’ coordinates. Thus, we may calculate a few non-physical subsonic speeds with artificially large variabilities, while the imperfections diminish. A similar overlap occurs at lower Mach numbers even with smooth shock fronts, yielding supersonic Mach numbers still with large variabilities. Using alternate metrics for averages and variabilities, such as means and standard deviations, respectively, does not yield significantly different results nor does it alleviate this artificial effect. Thus, while misrepresentative at low Mach numbers ($\lesssim 1.4$), the MAD does capture variability in velocity once the overlap in position PDFs diminishes.

### Focusing-shock trajectories

**Fig. 9:**
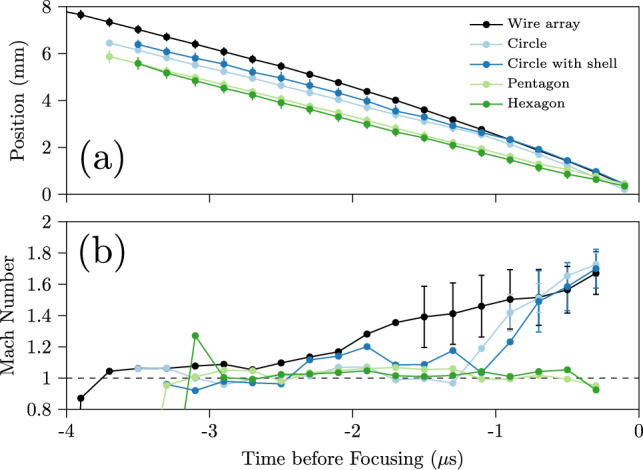
Time series of median **a** positions and **b** Mach numbers of shocks generated by the different foil geometries as well as the benchmark wire array. Quantified with the MAD, the variability in position is shown as error bars. The variability in velocity is similarly represented; however, we do not display MADs greater than 0.2 to keep the plot legible. The speed of sound in water is 1490 m/s

We present in Fig. 9 the position and velocity in time of the five different focusing shock geometries tested. Note first that the polygonal shocks travel at constant velocity, a behavior inconsistent with theoretical expectations presented in Sect. 1, but consistent with the above-mentioned observations of their constant shape, so we will explore this further in Sect. 4. In contrast, the circular shocks all accelerate to roughly Mach 1.7 close to focus regardless of which configuration generated them. Using the equation for pressure behind the shock (Zel’dovich and Raizer 2002)

2 $$\begin{aligned} P = \frac{2\rho _0U_S^2}{\gamma + 1}, \end{aligned}$$

where $\rho _0 = 998$ kg/m$^3$ is the initial water density, $U_S = 1.7\times 1490 = 2533$ m/s is the shock speed, and $\gamma \approx 7$ is the adiabatic coefficient of water, we estimate that the circular shocks generate pressures around 1.6 GPa. Self-similar solutions (Zel’dovich and Raizer 2002; Grinenko et al. 2007) relating pressure to shock radius can estimate the pressure even closer to focus, however, without additional insight (e.g. from simulations) as to when such solutions become invalid such estimates can grow unboundedly.

The wire array’s shock has an earlier, more gradual acceleration compared to that of the shocks created by the foils. Interestingly, the sudden accelerations of our foil-generated shocks around $-$1.2 $\mu$s resemble those observed in shocks from other wire-array explosions (Krasik et al. 2008; Bland et al. 2017). This acceleration is attributed to a secondary shock (created by the boiling, ionization, and explosion of the wires) catching up to a weak shock of Mach number $~\sim$1 that was initially launched by some pre-explosion process. However, were this the case, our wire-array and polygonal-foil explosion shocks should exhibit a similar acceleration, rather than gradually accelerating or not accelerating at all. This intriguing difference in shock trajectories would greatly benefit from further investigation, especially with the aid of simulations. Given this unresolved aspect, we do not to fit our trajectory data with an analytical model and only report results based on our statistical approach.

We repeated all tests at double the available electrical energy-per-mass by halving the foils’ length to 10 mm. Except for the free-standing circular foil, the main outcome was a serious deterioration in the quality and symmetry of the shocks. This is due to the increased difficulty in manufacturing such short foils without significant wrinkles. The shocks from the circular shell-attached and polygonal foils appear to travel at constant near-sonic speeds, whereas that from the free-standing circular foil still accelerated to about the same speed as its longer counterpart. Clearly, the deterioration in shape hindered the acceleration of the shell-attached foil’s shock, yet the free-standing circular foil’s shock seems to not have benefited from the additional available energy. This suggests that the energy was instead dumped into the plasma arc after the explosion instead of the expansion of the foil.

## Discussion

Our exploratory tests of shock generation with thin-foil explosions yielded initial promising results, particularly for the case of circular focusing shocks. Given the same available electrical energy-per-mass, the free-standing circular foil and the benchmark wire array produced symmetric shocks with similar Mach numbers close to focus (at least within our measurement limits). We also found that attaching the foil to the inside of a plastic shell did not affect the generated shock detrimentally. From an applied perspective, foils could then substitute wire arrays for underwater shock generation if concerns about the wires’ shocks fully coalescing exist (e.g., due to experimental spatial constraints). To build further confidence in the foil approach, future work should study the coupled dynamics of foil explosions and shock propagation, as is done with the exploding wire method (Grinenko et al. 2005; Krasik et al. 2016; Chung et al. 2016; Stephens et al. 2014; Hernández Garcia et al. 2025). This would help clarify why the trajectories of the foil and wire-array shocks differ, as well as provide valuable insight for improving the shock generation (as similarly done for wire arrays Fedotov et al. 2007; Bland et al. 2017).

More interesting to probe further now from a fundamental perspective is why the polygonal shocks do not behave as expected, even though they have sharp vertices and corrugations none worse than those in equivalent gas experiments (cf. Fig. 6 in Kjellander et al. (2010)). Instead of rounding, rotating, and accelerating due to the shock–shock interactions at their vertices, our polygonal shocks behave as independent planar shocks traveling at a constant near-sonic speed. This suggests that, though narrow (<1 mm, see Fig. 4c), the gaps in between the individual foil strips interfere with the shock–shock interactions, especially for such weak initial shocks. The alternative of using a single foil wrapped around the electrode adapters, as done with the circular foils, did not work as we could not properly tension the foil to have flat sides and sharp corners. However, considering the small (or at least non-detrimental) impact the plastic shell had on the circular shock’s behavior, testing a similar manufacturing approach with the polygonal foils may clarify why the shocks behave differently than expected.

**Fig. 10:**
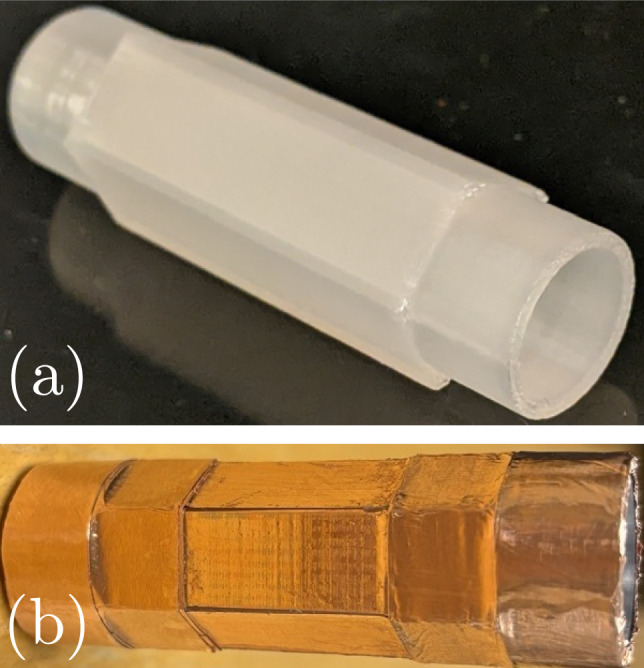
Hexagonal shell **a** without and **b** with copper tape and foil. The polygonal foils are still 20 mm long and have a circumscribing circle with a 20 mm diameter

To this end, we built two new configurations to place inside the test cell. The first was analogous to the cylindrical shell pictured in Fig. 3c but with a hexagonal cross section on the inside. We still glued six individual foil strips; however, now the shell helped us eliminate the gap between strips. While an improvement compared to the free-standing hexagon, aligning and attaching the strips without creating wrinkles remained challenging due to the awkward access to the inside of the shell. This motivated testing the second configuration pictured in Fig. 10. Here, we printed a hexagonal prism with thin walls ($\sim$1.5 mm) extending between the circular electrode adapters and glued the foil to the outside. With this design, we avoided wrinkling the foils and ensured truly flat sides. Yet we risked negatively impacting the uniformity of the foil explosions, unsuccessfully generating a shock in the plastic, or inadequately transmitting the shock to the water inside.

**Fig. 11:**
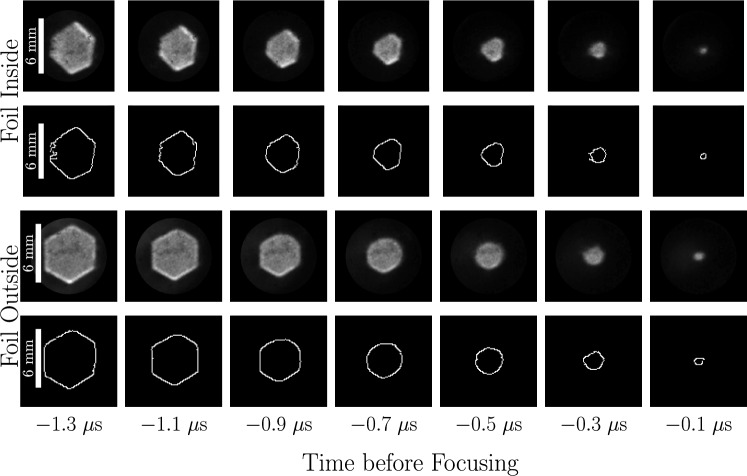
Time sequences of shadowgraph images (first and third rows) alongside the images’ detected edges (second and fourth rows) for the hexagonal focusing shock generated by the explosion of the copper foil placed on the inside and outside of a hexagonal shell, respectively. The rounding of the corners is evident for the outside foil and contrasts with the shock from the free-standing foil shown in Fig. 7

**Fig. 12:**
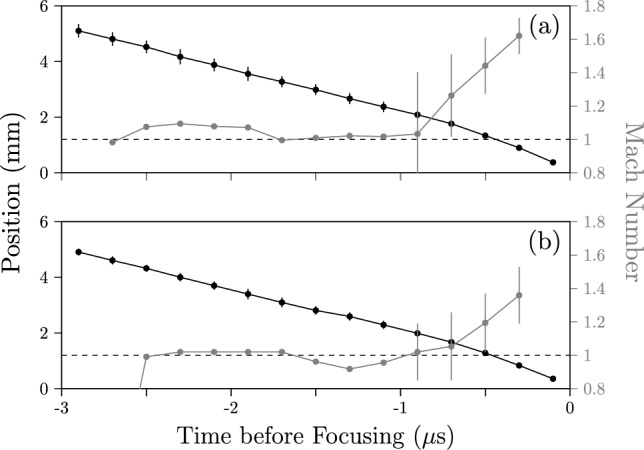
Time series of the median position and Mach number of the shock generated by the exploding foil attached to the outside of the hexagonal shell. Neither the **a** circular nor **b** polygonal definitions of position are adequate throughout the whole convergence due to the shock’s evolving shape; however, both indicate that the shock accelerates as it focuses

We present in Fig. 11 the sequences of shadowgraph images along with the corresponding detected edges for the focusing shocks created with these two shell-attached hexagonal foils. The discharge voltage was the same as the free-standing hexagon foil to keep the same available energy-per-mass. The voltage–current time traces of both discharges (not shown) do not differ significantly from those of the previous tests, again indicating that the presence of the plastic shell does not affect the foils’ explosion. Both foils exploded simultaneously about their circumference, and the shock’s initial hexagonal shape is slightly improved compared to the free-standing case. However, now this initial shape appears to evolve toward a circle as it focuses, as evidenced particularly by the frames 0.5 and 0.7 $\mu$s before focusing. This is more pronounced for the shock generated by the foil placed outside the shell, which is sensible given the higher manufacturing quality. Additionally, the measurements of this shock’s position and velocity shown in Fig. 12 indicate that an increase in shock speed accompanies this rounding of the shape. As the shock is neither circular nor hexagonal throughout the focusing, both definitions for position fail to properly characterize the shape’s trajectory, so the exact values are slightly misrepresentative. Despite this, the shock’s simultaneous rounding and acceleration starting around 0.7 $\mu$s before focusing clearly align with the expectations from theory (Schwendeman and Whitham 1987). This confirms our suspicions that the manufacturing quality of the polygonal foil design (particularly at the edges) indeed affects whether the individual shocks manage to interact with one another in the space and time available in our experiment.

Interestingly, the explosions of foils attached to the outside of analogous circular shells created less uniform shocks than those of the free-standing and inside shell-attached foils. The cause for this seems to be that, in the process of gluing the foil to the outside of the shell, noticeable wrinkles develop, especially down the length of the foil. This happened whether using one or multiple foils to cover the shell. Thus, while the polygonal shell’s flat surfaces greatly benefit the manufacturing process and the focusing shock’s shape, the same does not happen with the circular geometry’s convex surfaces. Potentially this is a manufacturing detail which can be remedied; however, foils attached to concave surfaces or left free standing already demonstrate good performance.

## Conclusion

In this paper, we explored how successful microsecond explosions of thin copper foils are at generating underwater focusing shocks of various geometries. We characterized the shocks generated by different foil configurations through statistical analysis of high-speed shadowgraph images, yielding the following main observations:

1. Using a PPG to deliver 250–350 kA in $\sim$1 $\mu$s, we exploded 10-$\mu$m-thick foils uniformly along their width and length (10–20 cm), directly producing planar or curved shocks with the desired 2D geometry. Minor corrugations in the shocks arise due to wrinkles present in the foils, yet these seem no more detrimental than similar imperfections in shocks generated by wire arrays.
2. In the circular geometries, shocks generated by a free-standing foil and a foil attached to the inside of a plastic shell exhibit similar trajectories and reach roughly the same Mach number close to focus. In comparison, the analogous shock generated by a benchmark wire array accelerates at a lower rate but for a longer time, thereby reaching the same Mach number close to focus.
3. While exhibiting decently flat sides and sharp corners, the shocks generated by free-standing polygonal foils do not undergo the rounding, rotation, and acceleration expected from theory (Schwendeman and Whitham 1987). We suspect the shock–shock interactions necessary to induce such behavior are compromised by their manufacturing quality. However, preliminary tests show that, if the foils are attached to a plastic shell, then the focusing shock indeed rounds and accelerates as expected.

Although seemingly surmounting manufacturing difficulties and providing a new avenue for studying shock focusing in water, the novel design shown in Fig. 10 spurs questions meriting investigation. How does a shock transmitted through the substrate differ from one created directly in the water? How do we self-consistently model the shock propagation through two different media? What properties should the substrate have, acoustic or otherwise, to produce an energy-efficient explosion and shock transmission? Existing research on single-foil explosions (Baksht et al. 2015; Shelkovenko et al. 2018, 2020a, b; Asmedianov et al. 2023) , the effect of strong shocks on solids (Rice et al. 1958; Duvall and Graham 1977; Asay and Shahinpoor 2012), and flyer acceleration (Chace and Moore 1962; Luo et al. 2013; Kim and Jang 2023; Asmedianov et al. 2025) can guide further studies of this new experimental configuration. In particular, determining whether the shock weakens or loses its symmetry as it transmits through the substrate would have important consequences for the extreme conditions produced by the focusing process.

## Data Availability

The data that support the findings of this study are openly available at https://doi.org/10.5281/zenodo.16563056.
